# Influence of the use of autogenous bone particles to close the access window after maxillary sinus floor augmentation: a micro‐computed tomography and positron emission tomography study in rabbits

**DOI:** 10.1007/s10006-022-01063-0

**Published:** 2022-04-28

**Authors:** Luigi Feletto, Daniele Botticelli, Karol Ali Apaza Alccayhuaman, Miguel Peñarrocha-Diago, Mustafa Ezzeddin-Ayoub, Regino Zaragozi-Alonso, Jose Viña-Almunia

**Affiliations:** 1 ARDEC Academy, Rimini, Italy; 2 Principal Research Scientist at ARDEC Academy, Rimini, Italy; 3 Department of Oral Biology, University Clinic of Dentistry, Medical University of Vienna, Vienna, Austria; 4 Oral Surgery Unit, Department of Stomatology, Faculty of Medicine and Dentistry, University of Valencia, Valencia, Spain; 5 Advanced Radiopharmaceutical Technician, Micro PET-CT Unit, Medical Central Investigation Unit (UCIM), Faculty of Medicine and Dentistry, University of Valencia, Valencia, Spain; 6 Oral Surgery and Implant Dentistry, Oral Surgery Unit, Department of Stomatology, Faculty of Medicine and Dentistry, University of Valencia, Valencia, Spain; 7 Oral Surgery Unit, Department of Stomatology, Faculty of Medicine and Dentistry, University of Valencia, C/Gasco Oliag 1, 46021 Valencia, Spain

**Keywords:** Sinus floor elevation, MicroCT, MicroPET, Xenograft, Osteoconductivity

## Abstract

**Aim:**

The purpose of this study was to evaluate using microCT and positron emission tomography (PET) analysis, the influence on bone healing of the placement of particulate autogenous bone in the antrostomy, and in the subjacent region after maxillary sinus elevation with xenograft.

**Material and methods:**

The sinus mucosa was elevated in sixteen male New Zealand rabbits and they were both grafted with a collagenated cortico-cancellous porcine bone. The antrostomy and the near subjacent region were filled with either the same xenograft (control site) or with particulate autogenous bone (test site) harvested from the tibia. The antrostomies were covered with collagen membranes. MicroCT (measured in Hounsfield Units) and microPET (kBq/cm^3^) using sodium fluoride infiltration (^18^F-NaF) were performed at the time of euthanasia that was performed after 1 and 8 weeks of healing, using 8 animals in each group. The Wilcoxon test was used for analysis.

**Results:**

At the microCT analysis, after 1 and 8 weeks of healing, no statistically significant differences were found between groups. Bone increased and xenograft decreased significantly between the two periods of healing. At the microPET analysis, the percentage of bone increased significantly over time in both test and control groups and no significant differences were found between groups.

**Conclusion:**

The placement of autogenous bone in the antrostomy and the subjacent region after maxillary sinus elevation did not enhance bone formation compared with sites where only xenograft was used. Both microCT and microPET showed increase bone formation over time.

## Introduction

Sinus floor augmentation is widely used by clinicians to allow implant installation in the posterior regions of an atrophic maxilla, providing optimal results [1]. A clinical study did not find a difference in survival rate for a lateral window with or without the protection of a membrane [2]. However, other clinical studies found higher amounts of new bone at sinuses at which the antrostomy was protected with a collagen membrane compared to unprotected sites [3, 4]. Nevertheless, a systematic review with meta-analysis did not find differences in bone formation between the two procedures [5]. It has been also shown that a collagen membrane was unable to avoid the extrusion of biomaterial through the antrostomy [6–8], so other methods, as repositioning the bone window on the antrostomy [9–12], or the use of non-resorbable membrane or ti-mesh have been suggested [6].

The histological evaluation represents the main method adopted for the analysis of the healing processes and present a high sensitivity in recognize tissues. The rabbit is a widely used model to study the healing after sinus augmentation and both histologic and microCT evaluations have been adopted in several studies [11–13]. The positron emission tomography (PET) might also be used to study bone formation [6]. PET imaging techniques are extensively employed clinically with radiopharmaceutical isotopes to detect tumors of osteogenic origin [15, 16]. Currently, this nuclear imaging technique has the potential to efficiently provide information on the chronological dynamics of bone response. The PET assessment uses 18F-ions, incorporated in the bone tissue trough a [18F]—sodium fluoride ([^18^F]-NaF) tracer. Tracer retention by the bone is a 2-phase process [17]. In the first phase, ^18^F_2_ OH_2_ exchanges for an ion on the surface of the hydroxyapatite matrix of bone. In the second phase, ^18^F_2_ migrates into the crystalline matrix of bone, where it is retained until the bone is remodeled. This way bone formation can be measured over time.

In a previous histomorphometric study with the same sample used in the present study, different regions of the elevated space were studied to evaluate the effect of the placement of autogenous bone closing the antrostomy after sinus elevation using a xenograft [18]. New bone formation was higher in the group where the antrostomy was closed with autogenous bone. However, a significant difference was only found in the subjacent region of the antrostomy. Further evaluation using different methods of analysis could provide additional useful information on bone regeneration. Thus, the aim of this study was to evaluate, using microCT and PET analyses, the influence on bone healing of the placement of particulated autogenous bone in the antrostomy and in the subjacent region after maxillary sinus elevation with xenograft.

## Material and methods

### Study design and experimental animals

Sixteen male New Zealand rabbits of about 24 weeks of mean age and 3–4 kg were used and divided in two groups of eight animals each, that were euthanized after either 1 or 8 weeks from surgery.

The sample of animals included in the present study was also used for histological analysis, the results of which had been published in another article [18]. In this study, the information will be reported in brief, while for details see the article mentioned above.

### Ethical statement

The protocol was approved by the Ethics Committee of Valencia University, Spain (A1434714637496) and the guidelines provided by the Council Directive of the European Union (53/2013; February 1, 2013) for animal experimentation and the ethical rules proposed by Royal Decrete 223, March 14 and October 13, 1988 were followed. The study was reported following the ARRIVE guidelines.

### Randomization and sample size

An author not involved in the surgery (DB) performed the randomization and the main surgeon (JVA) was informed about the allocation treatment after having grafted both sinuses, but before the filling of the antrostomy and the near subjacent region.

The sample size was based on the histological data from a study in rabbits and resulted in seven animals to be included each period of healing [18].

### Clinical procedures

**Fig. 1:**
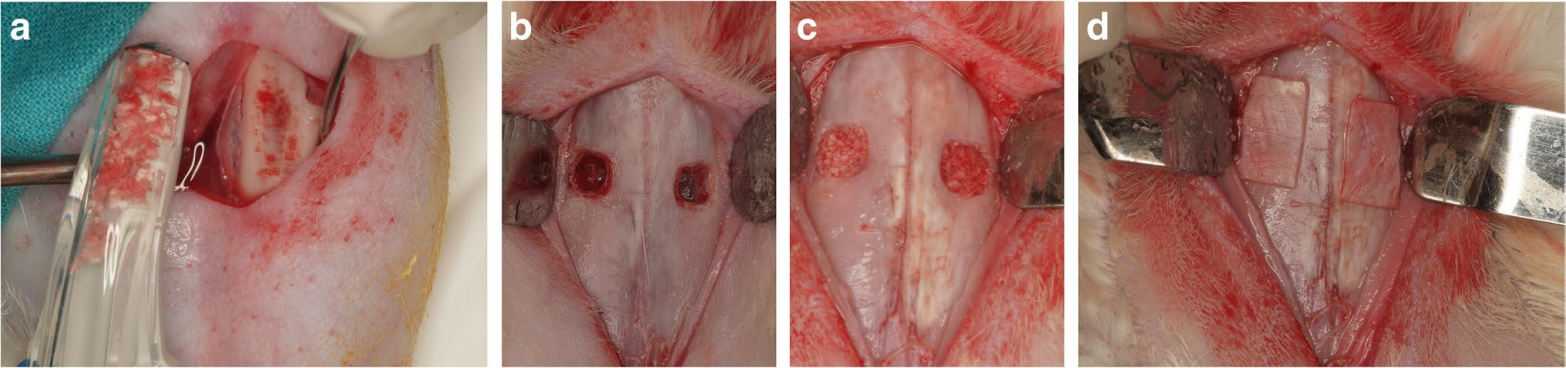
Surgical procedures. **a** Autogenous tibial bone particles were collected with a bone scraper **b** 4 × 4 mm antrostomies, located about 3–4 mm laterally to the midline, were prepared. **c** After sinus mucosa elevation, both sinuses were grafted with collagenated cortico-cancellous porcine bone (mmm). The antrostomy and the subjacent region were grafted with autogenous bone particles in the test group (left sinus of the image) or with the same xenograft in the control group (right sinus). **d** A collagen membrane (OsteoBiol® Evolution 0.3 mm, Tecnoss®, Giaveno, Italy) was used to cover both antrostomies

Briefly (for details see Favero et al. 2020 [18]), after having provided general anesthesia and shaved the experimental regions, autogenous bone was collected from the anterior tibia tuberosity using a Savescraper Twist curve (CGM spa, Divisione Medicale META, Reggio Emilia, Italy) and maintained in saline (Fig. 1a).

Subsequently, two 4 × 4 mm in dimensions antrostomies were prepared on the nasal dorsum, one each side laterally to the nasal incisal suture, and about 10 mm in front of the nasal-frontal suture (Fig. 1b). After elevation of the sinus mucosa with a small elevator (Bontempi®, San Giovanni in Marignano, RN, Italy), the space was grafted with ~ 125 mg of collagenated cortico-cancellous porcine bone (OsteoBiol Gen-Os, Tecnoss®, Giaveno, Italy; 250–1000 μm). The antrostomies and the subjacent region were randomly grafted with either the same xenograft (control sites) or with particulated bone (test sites) (Fig. 1c). A collagen membrane (OsteoBiol® Evolution 0.3 mm, Tecnoss®, Giaveno, Italy) was used to cover both antrostomies (Fig. 1d).

The animals received analgesics and anti-inflammatory drugs and were maintained in individual cages in acclimatized rooms at the animal facilities of the University of Valencia. Wound and health conditions were monitored for the whole experimental period by professionals.

### ^18^F-Sodium fluoride injection

The rabbits were, the day before of the infiltration of ^18^F-NaF, weighed (5.71** ± **1.17) to adjust the dosage to the (based on) weight of the rabbit. Before euthanasia, a dose of 0.565 ± 0.167 mCi of ^18^F-NaF radiotracer, developed at Advanced Accelerator Applications (Iberica S.L. Esplugues de Llobregat, Barcelona, Spain), was intravenously applied through injection into the ear. The ^18^F-NaF was allowed to rest for 35 min in the animal’s body before PET analysis.

### Euthanasia

The animals were euthanized using 50 mg/kg of sodium pentobarbital (Nembutal® Schaumburg, IL, USA). Subsequently, the maxilla was separated from the rest of the skull and a microCT-PET scan was performed.

### MicroCT/MicroPET

The biopsies were scanned in a high-resolution hybrid microPET/CT (Albira I, Bruker Biospin PCI GmbH Rheinstetten, Germany).

This microCT is equipped with a tungsten anodic tube with a maximum voltage of 50 Kvp (50 W) and 1.0 mA, and a focal spot size tube of 35 μm. The spatial resolution is 70 μm. The sensor is a scintillator coupled to a bidimensional array photodiode. The exposure was realized using a high voltage (45 kV) and high intensity (0.8 mA) system and lasted for 7 min.

The PET works through a radiotracer that contains a radioactive isotope (^18^F-NaF) that emits positrons. When positrons met its antiparticle, the phenomenon of annihilation occurs, and two photons are emitted (511 keV) in the opposite direction forming a 180 degree angle where finally they are collected by the detector. The microPET has a spatial resolution of 1 mm. The images were processed and reconstructed by Albira Reconstructor (Rheinstetten, Germany) (OSEM cross algorithm that allows 2D and 3D image reconstruction). Data acquisition was performed following a predefined protocol with Albira Acquirer from New Albira Suite 3.0 software (Rheinstetten, Germany).

**Fig. 2:**
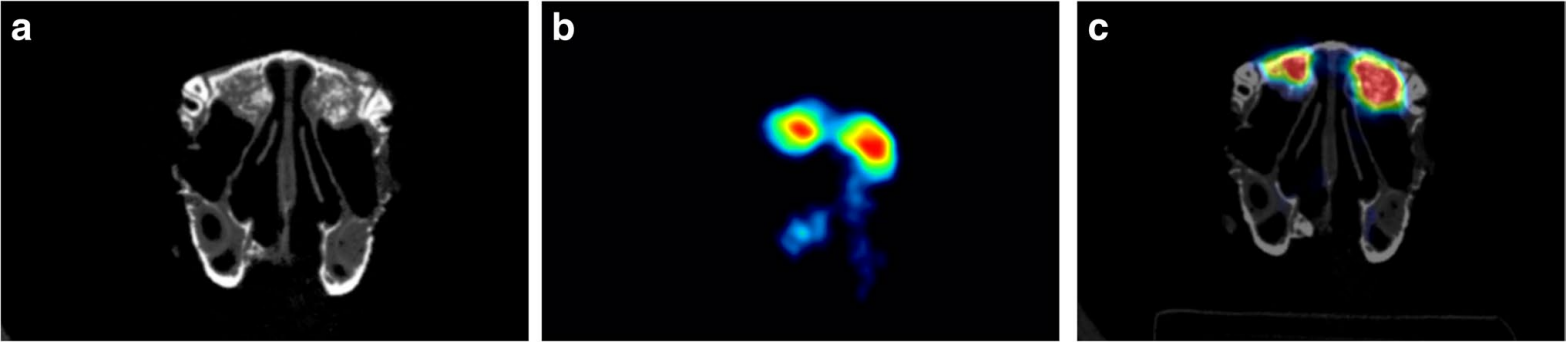
Coronal plane of both radiological evaluations. **a** Micro-CT images. Both sinuses were grafted and different thresholds of Hounsfield units (HU) levels were used to assess bone healing. **b** PET analysis. PET assessment was measured in kBq/cm^3^. 18F-NaF captation was represented by a color scale and **c** Integrated microCT and PET images which allowed a comparative analysis of both radiological studies

For microCT image, a full body image was done in 7 min, while for the microPET image, a 2-frame full body image was obtained in 15 min. The cross-sectional images were processed and reconstructed in order to be visualized. They were subsequently analyzed using the algorithm CT Rec. Alg. for the CT (Fig. 2a), and OSEM Cross Alg. for PET (Fig. 2b) with Albira Reconstructor (New Albira Suite 3.0 software). Images were visualized through PMOD software (PMOD Technologies® LLC, Zurich, Switzerland). The Integrated microCT and PET images allowed a comparative analysis of both radiological studies (Fig. 2c).

### Volume of interest

**Fig. 3:**
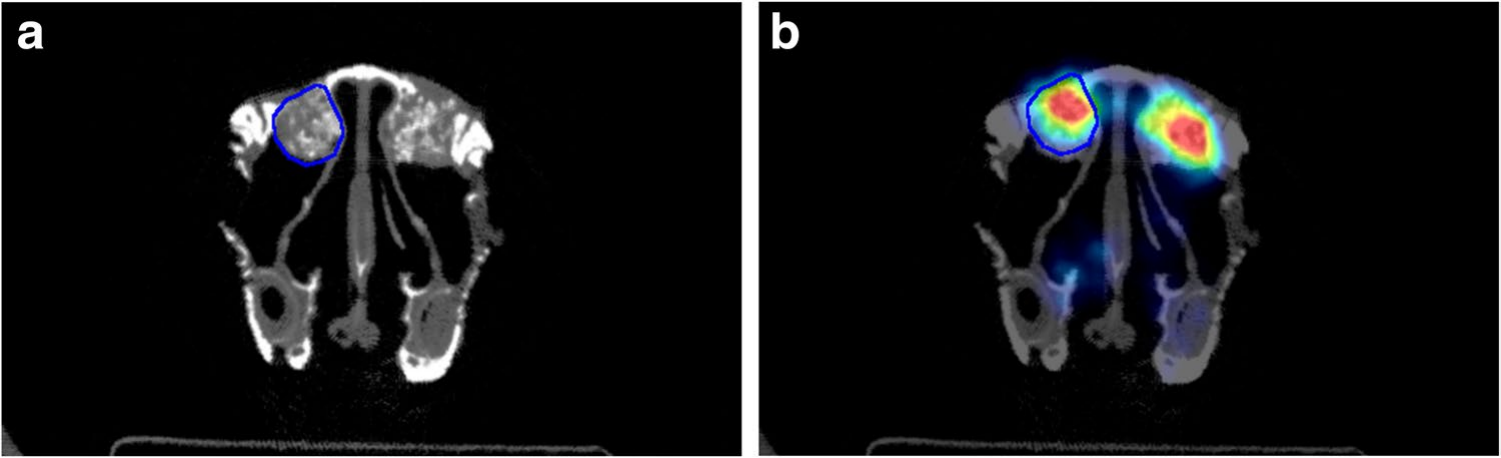
**a** Volume of interest (VOI) performed in the micro-CT through a PMOD software (PMOD Technologies® LLC, Zurich). Once the VOI was design, **b** a superimposition of the micro-CT and microPET was performed for the assessment of both radiological studies. So, in order to avoid bias, the same VOI was used in the micro-CT and microPET

The volume of interest (VOI) was performed in the microCT scan through PMOD software (PMOD Technologies® LLC, Zurich, Switzerland). The assessed VOI was a region of 2 mm anteriorly and posteriorly (4 mm in total) to the center of the osteotomy in the anterior–posterior plane of the augmented sinus. Subsequently, the cross sections were interpolated to obtain all VOIs (Fig. 3a). Once the VOI was defined in each maxillary sinus, an overlap with the PET image was done in order to obtain the same VOI in both microCT and the PET assessments (Fig. 3b).

### MicroCT and PET assessment

In order to discern bone from xenograft in the microCT evaluation, different thresholds of Hounsfield units (HU) levels were used in each time point. The thresholds of grey levels for both new bone and xenograft were identified based on previous studies [19] and on the visual analysis of the images. To allow a comparison of data between periods, similar thresholds were selected for 1- and 8-week periods. PET assessment was measured in kBq/cm^3^ i.e., the osseous metabolic activity by cm^3^.

### Statistical analysis

Mean values and standard deviations were calculated for each outcome variable. The software Excel 2013 (Microsoft Corporation, Redmond, WA, USA) was sued to perform all calculations. The Wilcoxon rank-sum test was applied for testing differences using the software IBM SPSS Statistics software v.19 (IBM Inc., Chicago, IL, USA).

## Results

Technical problems with image acquisition did not allow to obtain data of two rabbits of the 1-week group, so the total sample was *n* = 6 and *n* = 8 for the 1-week and 8-week groups, respectively.

### PET analysis

**Fig. 4:**
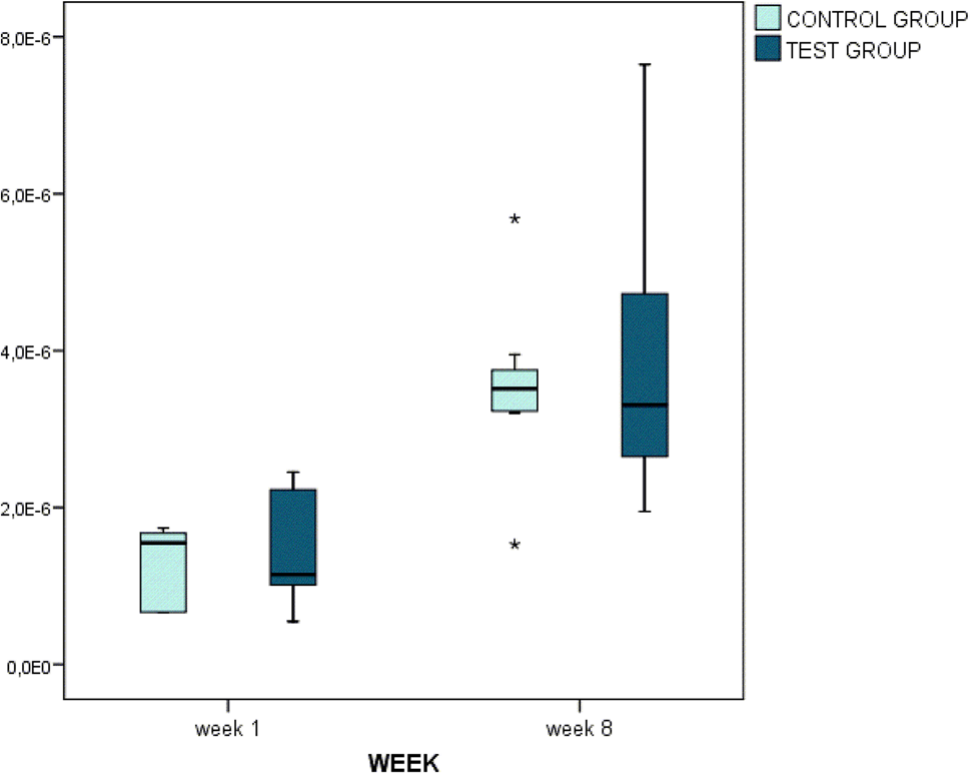
PET analysis. KBq/cm^3^ at one and 8 weeks post-surgery. Note that there was an increase in PET analysis between both times of measurement (*p* < 0.001). There were no statistically significant differences between groups in each time of healing

Considering the whole sample, there was a significant increase in uptake (*p* < 0.001) in the kBq/cm^3^ between 1 and 8 weeks. That increase over time was similar in both groups (1.55 × 10^−6^ to 3.52 × 10^−6^ kBq/cm^3^ in the control group, and 1.15 × 10^−6^ to 3.31 × 10^−6^ kBq/cm^3^ in the test group) (*p* = 0.854). There were no significant differences between groups either at one (*p* = 1.000) and at 8 weeks of healing (*p* = 0.782) (Fig. 4).

### CT analysis

**Fig. 5:**
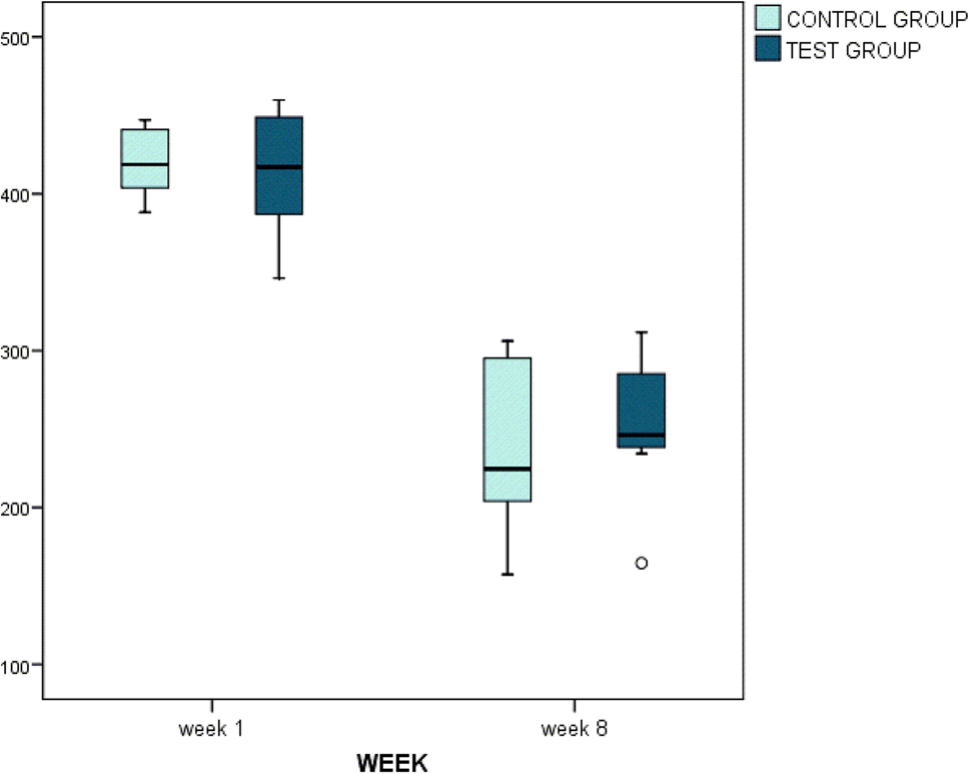
CT analysis. The figure shows a decrease in the HU between 1 and 8 weeks of healing (*p* < 0.001). The decrease was similar in both groups (*p* = 0.410)

Table 1 describes CT analysis data at 1 and 8 weeks of healing in both groups. Considering the whole sample, there was a decrease over time in the HU (1 to 8 weeks of healing) that reached statistically significance (*p* < 0.001). Both groups experimented a similar decrease (*p* = 0.410). Figure 5 also shows the decrease of HU over time in both groups.

**Table 1:** CT analysis expressed in HU for the total sample. There was a decrease in HU values between both times of measurement. Analyzing the effect of the graft used, results showed that there were similar HU values between groups

	**Week 1**	**Week 8**	***p*-value (week 1 vs.8)**	***p*-value (B-L model)**
**Control group**	418.7	224.7	0.001**	Week effect < 0.001***
**Test group**	416.9	246.2	0.001**	Graft effect: 0.549
***p*-value (control group vs. test group)**	0.877	0.257		Interaction: 0.410

^*^p < 0.05; **p < 0.01; ***p < 0.001

At 1 week of healing, there were no differences between groups. New bone fraction was 4.9 ± 3.6% and 4.4 ± 1.7% for the test and control sites, respectively (*p* = 0.600). The respective biomaterial fractions were 55.8 ± 14% and 57 ± 7.5% (*p* = 0.600) (Table 2).

**Table 2:** CT analysis. Percentages (%) of bone and xenograft in each VOI for each period of time. Note that there was an increase of bone between 1 and 8 weeks while the percentage of xenograft decreased. Analyzing the effect of the graft used, results showed similar values between groups

		**One week**	**Eight weeks**
**Test sites**	Bone	4.9 ± 3.6	25.2 ± 10.5
	Xenograft	55.8 ± 14.0	13.0 ± 8.8
**Control sites**	Bone	4.4 ± 1.7%	24.0 ± 11.4
	Xenograft	57.0 ± 7.5	11.7 ± 6.0

After 8 weeks of healing, new bone increased to 25.2 ± 10.5% and 24 ± 11.4% at the test and control sites, respectively (*p* = 0.401). The biomaterial, conversely, decreased in both groups to 13.0 ± 8.8% and to 11.7 ± 6% in the test and control sites, respectively.

## Discussion

The present experiment aimed to study the influence on the healing of the placement of autogenous bone on the antrostomy and in the subjacent region after maxillary sinus elevation with xenograft. Two radiological examinations were performed: a microCT and a PET. The results of the present study showed that both radiological (imaging) methods were in accordance. Both, the microCT-PET showed an increase in bone formation between 1 and 8 weeks of healing, and similar results in terms of bone formation were found in both graft approaches.

The present experiment reported the radiological evaluation of a previous histomorphometric study recently published using the same sample of animals [18]. Similar to the present, the histomorphometric study showed increased bone formation over time in both groups and, conversely, a reduction of the proportions of the biomaterial. Differences in proportions were observed between histological and microCT evaluations. After 1 week, the microCT revealed in both groups 4–5% of new bone while, in the histological assessment, the proportion was of ~ 3%. The biomaterial proportion in the present experiment was ~ 56–57% while in the histological evaluation ~ 42% in the test sites, and ~ 53% in the control sites. After 8 weeks of healing, bone increased to ~ 25% in the test sites, and to 24% in the control sites. The corresponding proportions evaluated histologically were ~ 28% and ~ 24%. The biomaterial decreased in both evaluations, to 12–13% at the microCT analysis, and to ~ 17% at the histological analysis.

These differences between histologic and tomographic studies using the same sample are reported in the scientific literature [20, 21]. In a similar experiment in rabbits using the same brand of xenograft used in the present study [21], biopsies from sinus augmented with xenograft were analyzed both at the microCT and histologically. Especially after 2 weeks, statistically significant differences were found between the two methods of measurements. At the histological study, new bone was represented by about 8%, while at the microCT analysis was from 12 to 18% depending on the level of grey threshold used. Xenograft as well presented statistically significant difference at the 2-week period, being about 32% at the histological assessment, and 18–21% at the microCT evaluation. One reason that could explain the differences between histological and tomographic analysis in the present study is the similarity between density and the mineral content of bone and the xenograft used. In an in vitro study [22], the density and mineral content of the xenograft used were 2.43 g/cm^3^ and 64.6%, and for and natural human bone was 2.30 g/cm^3^ and 65.0%, facts that make difficult the discrimination between xenograft and bone.

Accordingly, the data from the microCT analysis, also the microPET assessment showed increase bone formation between one to 8 weeks of healing, without notable differences between the two graft modalities. There are few studies assessing the healing using biomaterials for bone augmentation procedures in dentistry using microCT/PET technology. For instance, in an experimental study in rats, the healing in calvaria critical-sized defects filled with different biomaterials were analyzed with a microCT/PET analysis [14]. Their results are difficult to compare with the present ones because graft materials, follow-up time points and preclinical model differs from the present study. In another experimental study in rats, the effect of immediately loaded implants as also evaluated using microCT/PET [23, 24]. The authors concluded that Na18F-PET allowed estimate of bone metabolism changes around the implant [23, 24].

Within the limits due to the preclinical nature, this study suggests that the placement of autogenous bone in the antrostomy and the subjacent region after maxillary sinus elevation did not contribute to enhanced bone formation compared with sites only grafted with xenograft. Both, microCT and microPET analyses, showed increase bone formation over time and were effective in detecting new bone formation and biomaterial resorption.

## Data Availability

The datasets used or analyzed during the current study are available from the corresponding author on reasonable request.

## Abbreviations

PET: Positron emission tomography
mm: Millimeters
mg: Milligrams
mCi: Millicurie
kg: Kilograms
Kvp: Peak kilovoltage
W: Watt
μm: Nanometer
kV: Kilovolt
mA: Milliampere
KeV: Kilo-electronvolt
kBq: Kilobecquerel
cm^3^: Cubic centimeter
HU: Hounsfield units
