# Correction to: Influence of the use of autogenous bone particles to close the access window after maxillary sinus floor augmentation: a micro-computed tomography and positron emission tomography study in rabbits

**DOI:** 10.1007/s10006-022-01077-8

**Published:** 2022-06-02

**Authors:** Luigi Feletto, Daniele Botticelli, Karol Ali Apaza Alccayhuaman, Miguel Peñarrocha-Diago, Mustafa Ezzeddin-Ayoub, Regino Zaragozi-Alonso, Jose Viña-Almunia

**Affiliations:** 1 ARDEC Academy, Rimini, Italy; 2 Principal Research Scientist at ARDEC Academy, Rimini, Italy; 3 Department of Oral Biology, University Clinic of Dentistry, Medical University of Vienna, Vienna, Austria; 4 Oral Surgery Unit, Department of Stomatology, Faculty of Medicine and Dentistry, University of Valencia, Valencia, Spain; 5 Advanced Radiopharmaceutical Technician, Micro PET-CT Unit, Medical Central Investigation Unit (UCIM), Faculty of Medicine and Dentistry, University of Valencia, Valencia, Spain; 6 Oral Surgery and Implant Dentistry, Oral Surgery Unit, Department of Stomatology, Faculty of Medicine and Dentistry, University of Valencia, Valencia, Spain; 7 Oral Surgery Unit, Department of Stomatology, Faculty of Medicine and Dentistry, University of Valencia, C/Gasco Oliag 1, 46021 Valencia, Spain

**Correction to: Oral and Maxillofacial Surgery (2022)**

10.1007/s10006-022-01063-0

Originally, the article has been published online with error in author name. Author Karol Apaza-Alcayhuaman should be replaced with Karol Ali Apaza Alccayhuaman.

The original article has been corrected.

